# Mitophagy in Diabetic Cardiomyopathy: Roles and Mechanisms

**DOI:** 10.3389/fcell.2021.750382

**Published:** 2021-09-27

**Authors:** Haoxiao Zheng, Hailan Zhu, Xinyue Liu, Xiaohui Huang, Anqing Huang, Yuli Huang

**Affiliations:** ^1^Department of Cardiology, Shunde Hospital, Southern Medical University (The First People’s Hospital of Shunde), Foshan, China; ^2^Guangdong Provincial Key Laboratory of Shock and Microcirculation Research, Guangzhou, China; ^3^The George Institute for Global Health, Faculty of Medicine, University of New South Wales, Sydney, NSW, Australia

**Keywords:** diabetic cardiomyopathy, mitochondrial quality control, mitophagy, mitochondrial biogenesis, mitochondrial dynamics

## Abstract

Cardiovascular disease is the leading complication of diabetes mellitus (DM), and diabetic cardiomyopathy (DCM) is a major cause of mortality in diabetic patients. Multiple pathophysiologic mechanisms, including myocardial insulin resistance, oxidative stress and inflammation, are involved in the development of DCM. Recent studies have shown that mitochondrial dysfunction makes a substantial contribution to the development of DCM. Mitophagy is a type of autophagy that takes place in dysfunctional mitochondria, and it plays a key role in mitochondrial quality control. Although the precise molecular mechanisms of mitophagy in DCM have yet to be fully clarified, recent findings imply that mitophagy improves cardiac function in the diabetic heart. However, excessive mitophagy may exacerbate myocardial damage in patients with DCM. In this review, we aim to provide a comprehensive overview of mitochondrial quality control and the dual roles of mitophagy in DCM. We also propose that a balance between mitochondrial biogenesis and mitophagy is essential for the maintenance of cellular metabolism in the diabetic heart.

## Introduction

Diabetes mellitus (DM) is one of the most common chronic diseases and now places a substantial burden on public health worldwide. There are ∼451 million patients with DM worldwide and it is predicted that this number will rise to 693 million by 2045 (Cho et al., 2018). Cardiovascular complications are the leading cause of mortality associated with DM, accounting for 50–80% of deaths (Rawshani et al., 2017). Diabetic cardiomyopathy (DCM) is a non-ischemic and non-hypertensive cardiomyopathy that is caused by diabetic metabolic disorders (Maack et al., 2018). Early DCM is characterized by diastolic dysfunction and left ventricular hypertrophy, and systolic dysfunction develops in the middle or late stages of DCM, and can be associated with myocardial fibrosis and apoptosis (Marwick et al., 2018).

**FIGURE 1:**
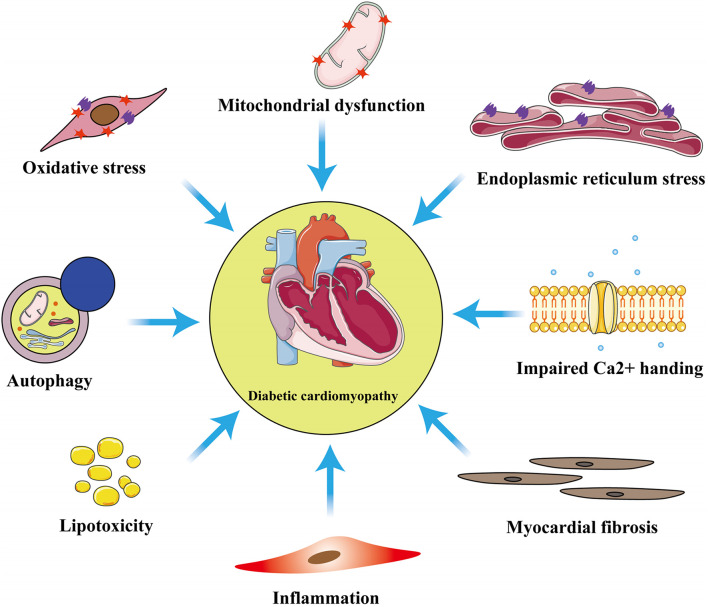
Principal pathophysiological mechanisms in diabetic cardiomyopathy. The exact pathogenesis of diabetic cardiomyopathy (DCM) remains unclear, but numerous studies have shown that these pathological processes: inflammation, myocardial fibrosis, oxidative stress, mitochondrial dysfunction, endoplasmic reticulum stress, autophagy, lipotoxicity, and impaired calcium homeostasis are involved in the development of DCM.

Although the exact pathophysiologic mechanisms of DCM have not yet been fully characterized, mitochondrial dysfunction, oxidative stress, inflammation, cardiomyocyte apoptosis or necrosis, autophagy, endoplasmic reticulum stress, myocardial fibrosis, and lipotoxicity are all involved (Figure 1). Among these mechanisms, mitochondrial dysfunction makes a substantial contribution to diabetic myocardial metabolic disorders (Wu S. et al., 2019).

Myocardial energy substrate use is adjusted to meet the high energy demands of the heart. Fatty acid oxidation is the principal contributor to ATP production (40–60%) and carbohydrate metabolism generates the remainder (20–40%) (Karwi et al., 2018). However, DM is characterized by insulin deficiency and/or insulin resistance, which lead to a reduction in glucose metabolism and an impairment in “substrate flexibility,” such that lipid metabolism increases in the diabetic heart. This impairment in mitochondrial fatty acid oxidation leads to the accumulation of fatty acids and lipid droplets in cardiomyocytes (Drosatos and Schulze, 2013). Furthermore, mitochondrial dysfunction caused by oxidative stress contributes to the development of DCM (Garcia-Touza and Sowers, 2012). Therefore, mitochondrial quality control may represent a means of reducing cardiac injury in diabetes (Kobayashi and Liang, 2015).

Mitochondrial fusion and fission (mitochondrial dynamics), mitochondrial biogenesis, and mitophagy are important components of mitochondrial quality control. Thus, disordered mitochondrial dynamics in diabetic cardiomyocytes, as well as an imbalance between mitophagy and mitochondrial biogenesis, may contribute to the development of DCM (Liang and Kobayashi, 2016). In this review, we focus on the roles and mechanisms of mitophagy in DCM. Mitophagy is a key component in mitochondrial quality control, and therefore it is often inseparable from mitochondrial dynamics and mitochondrial biogenesis. We will thus also discuss the interactions of these processes in DCM. Finally, we will summarize the potential therapeutic targets for mitophagy in DCM.

## Mitochondrial Dysfunction in Diabetic Cardiomyopathy

Mitochondria are referred to as the “power houses” of the cell and are double-membrane organelles that contain their own genome (Ernster and Schatz, 1981). Their principal function is to generate energy in the form of ATP by oxidative phosphorylation. Mitochondria play essential roles in various physiologic and pathologic processes, such as apoptosis, aging, autophagy, the production of reactive oxygen species (ROS), intracellular calcium homeostasis, and the metabolism of amino acids, lipids, and glucose (Vakifahmetoglu-Norberg et al., 2017). The heart is a highly active organ that has a large energy requirement. It is estimated that the adult human heart generates and consumes kilogram quantities of ATP daily to maintain the circulation. Mitochondrial oxidative phosphorylation is the major source for almost all the ATP generated (>95%) in the adult mammalian heart (Ashrafian et al., 2007), and 60% of the energy consumed by the heart comes from the oxidation of fatty acids in mitochondria (Vasquez-Trincado et al., 2016). However, in type 2 diabetes mellitus (T2DM), insulin resistance leads to lower glucose utilization and oxidative reduction, which means that the cardiomyocytes become almost completely dependent on the energy supplied by fatty acid oxidation.

These changes in the substrates used to generate energy result in an imbalance in the uptake and oxidation of fatty acids, which leads to mitochondrial dysfunction (Jia et al., 2018). Furthermore, hyperglycemia induces mitochondrial oxidative stress and mitochondrial fragmentation, which can cause cellular injury and dysfunction (Yu et al., 2006). Mitochondria occupy approximately 30% of the volume of cardiomyocytes and are the principal source of ROS (He et al., 2014). In diabetes, progressive mitochondrial impairment in cardiomyocytes causes lipid accumulation and results in the generation of a large amount of ROS, which increase oxidative stress, worsening the DCM and further impairing myocardial function (Volpe et al., 2018). Therefore, an effective treatment would be to remove or repair the impaired mitochondria and generate new mitochondria to maintain a pool of healthy mitochondria, which represents mitochondrial quality control. Next, we will elaborate on the roles and mechanisms of mitophagy in DCM and the interactions among mitophagy, mitochondrial dynamics, and mitochondrial biogenesis in DCM.

## Mitophagy in Diabetic Cardiomyopathy

The removal of metabolic waste products is an essential component of homeostasis. To prevent the accumulation of toxic molecules, make room for the addition of new elements, or reuse structures, organisms have developed complex systems to degrade and clear substances that are no longer needed (Dikic, 2017). Autophagy, which is also referred to as “macroautophagy,” is a degradation system that catabolizes cellular components, such as the cytosol, organelles, and protein aggregates, through the generation of autophagosomes (Yang and Klionsky, 2010). Selective autophagy is used to remove metabolic waste: specific substrates, such as unfinished or damaged protein complexes, or entire subcellular structures, are degraded by lysosomes (Gatica et al., 2018). Mitophagy, the best characterized type of selective autophagy, is the process whereby damaged or unwanted mitochondria are specifically degraded (Galluzzi et al., 2017). This process is particularly important for cardiovascular homeostasis and the protection of the myocardium in cardiovascular diseases, including myocardial infarction, cardiac hypertrophy, heart failure, ischemia/reperfusion, and DCM (Bravo-San et al., 2017; Morales et al., 2019).

**FIGURE 2:**
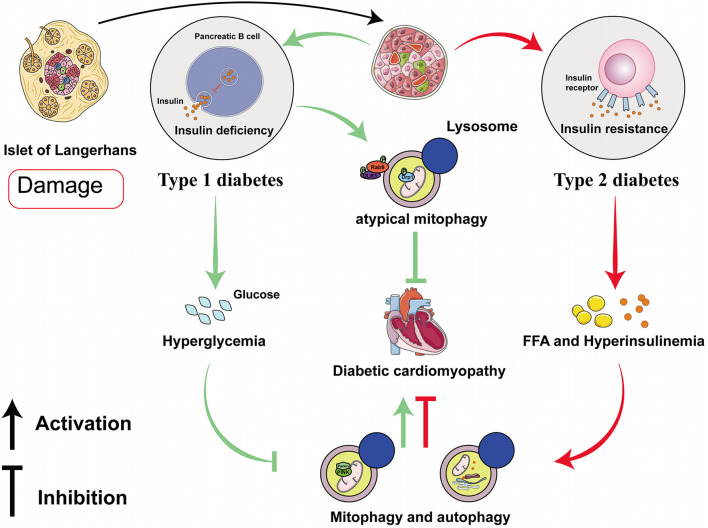
Roles of mitophagy and autophagy in diabetic cardiomyopathy in type 1 and type 2 diabetes mellitus. In diabetic cardiomyopathy (DCM) in T1DM, autophagy and PINK/parkin-mediated mitophagy are impaired, but atypical mitophagy is activated to limit cardiac damage. In DCM in T2DM, both autophagy and mitophagy are activated in the early stages, and they play compensatory roles that protect the myocardium.

Recent studies have suggested that mitophagy plays a protective role in DCM, principally through the clearance of abnormal mitochondria, which prevents oxidative stress and reduces myocardial apoptosis. Interestingly, the results and mechanisms of mitophagy may differ in patients with type 1 diabetes mellitus (T1DM) and T2DM (Figure 2 and Table 1). The etiology of T1DM is largely genetic and involves autoimmunity, which causes the loss of insulin-secreting pancreatic β-cells. In contrast, T2DM is characterized by insulin resistance (van Belle et al., 2011). The changes involved in mitophagy in individuals with T1DM and T2DM have not been fully characterized. Previous studies have suggested that insulin inhibits autophagy, but a deficiency in insulin substrate receptor 1/2 (IRS1/2) prevents the inhibitory effect of insulin on neonatal autophagy (Riehle et al., 2013). Therefore, autophagy would be expected to be activated in T1DM because of insulin deficiency. However, most previous studies have shown that autophagy and mitophagy are suppressed in the hearts of animal models of T1DM (Xie et al., 2011; He et al., 2013; Tang et al., 2015; Yu et al., 2017; Zhang M. et al., 2017; Wang et al., 2018, 2019; Xiao et al., 2018; Kobayashi et al., 2020). For example, Xu et al. (2013) showed that autophagy is inhibited in the early stages of DCM in mice with T1DM, whereas mitophagy is inhibited in the later stages. The authors also found that the myocardium of mice with T1DM expresses low levels of PTEN-induced kinase 1 (PINK1) and parkin, but high levels of the small GTPase RAB9, which suggests that the cardiomyocytes of these mice may undergo atypical mitophagy (Xu et al., 2013). Therefore, they proposed that when typical mitophagy, mediated *via* the PINK1/parkin pathway, is suppressed in T1DM, an alternative mechanism of mitophagy operates. Saito et al. (2019) found that this atypical mitophagy can protect the heart against ischemia through the ULK-RAB9 pathway. However, the mechanisms of RAB9-dependent mitophagy in type 1 DCM remain to be more fully characterized.

**TABLE 1:** The roles of auto/mitophagy in Type 1 DCM and Type 2 DCM.

Type of DM	Model	Intervention for DM *in vitro* or *vivo*	Changes of auto/mitophagy	Roles of target signals on auto/mitophagy	Effects on cardiac function	References
T1DM	*In vitro*: H9c2 cell *In vivo*: FVB mice	*In vitro*: HG (30 mM) *In vivo*: STZ (50 mg/kg)	Autophagy inhibition	AMPK-Beclin1-Bcl2 activated autophagy	Reduced CMs apoptosis	He et al., 2013
T1DM	OVE26 mice	STZ (50 mg/kg)	Autophagy inhibition	AMPK activated autophagy	Reduced CMs apoptosis	Xie et al., 2011
T1DM	*In vitro*: NMCMs *In vivo*: C57BL/6 mice	*In vitro*: HG (NA) *In vivo*: STZ (50 mg/kg)	Autophagy inhibition	Melatonin-Mst1-Sirt3 activated autophagy	Alleviated cardiac dysfunction	Zhang M. et al., 2017
T1DM	*In vitro*: NRCMs *In vivo*: C57/B6 mice	*In vitro*: HG (33 mM) *In vivo*: STZ (50 mg/kg)	Autophagy inhibition	TAX1BP1- NF-κB activated autophagy	Alleviated cardiac hypertrophy and fibrosis	Xiao et al., 2018
T1DM	*In vitro*: NMCMs *In vivo*: C57/B6 mice	*In vitro*: HG (30 mM) *In vivo*: STZ (50 mg/kg)	Auto/mitophagy inhibition	Sirt3-Foxo3A-Parkin activated auto/mitophagy	Reduced CMs apoptosis	Yu et al., 2017
T1DM	*In vitro*: NMCMs *In vivo*: C57BL/6 mice	*In vitro*: HG (33 mM) *In vivo*: STZ (50 mg/kg)	Mitophagy inhibition	Mst1-Sirt3-parkin inhibited mitophagy	Induced cardiac injury	Wang et al., 2019
T1DM	*In vitro*: NMCMs *In vivo*: C57BL/6 mice	*In vitro*: HG (NA) *In vivo*: STZ (50 mg/kg)	Mitophagy inhibition	Melatonin-Mst1-parkin activated mitophagy	Alleviated cardiac dysfunction	Wang et al., 2018
T1DM	*In vitro*: NRVCs *In vivo*: mt-Rosella mice	*In vitro*: HG (30 mM) *In vivo*: STZ (50 mg/kg)	Mitophagy inhibition	NA	NA	Kobayashi et al., 2020
T1DM	C57BL/6 mice	STZ (150 mg/kg)	Autophagy inhibition Mitophagy activation	Beclin1 activated autophagy Rab9 activated mitophagy	Induced cardiac injury Limited cardiac injury	Xu et al., 2013
T2DM	*In vitro*: NRCMs *In vivo*: db/db mice	*In vitro*: HG (40 mM) + Ole (200 μM)/Pal (200 μM)	Mitophagy inhibition	H2S-USP8-parkin activated mitophagy	Improved cardiac function	Sun et al., 2020
T2DM	*In vitro*: NMCMs *In vivo*: C57BL/6J	*In vitro*: Pal (200 μM) *In vivo*: HFD (NA)	Mitophagy inhibition	JQ1-BRD4- PINK1-parkin activated mitophagy	Alleviated cardiac dysfunction	Mu et al., 2020
T2DM	SD rat	HFD (45%) + STZ (40 mg/kg)	Mitophagy inhibition	Sirt6-AMPK-PGC1α-AKT activated mitophagy	Alleviated cardiac dysfunction	Yu et al., 2021
T2DM	C57Bl/6 mice	Fructose (60%)	Autophagy activation	Fructose	Induced cardiac remodeling	Mellor et al., 2011
T2DM	*In vitro*: AFCMs *In vivo*: C57BL/6 mice	*In vitro*: SFA (1.5 mM myristate or 2.0 mM Pal) *In vivo*: HFD (60%)	Autophagy activation	Ceramide synthase 5	Induced cardiac hypertrophy	Russo et al., 2012
T2DM	*In vitro*: AMCMs *In vivo*: C57BL/6 mice	HFD (60%)	Autophagy inhibition Mitophagy activation	Tat-Beclin1 activated autophagy	Alleviated cardiac hypertrophy, diastolic dysfunction	Tong et al., 2019

*DCM, diabetic cardiomyopathy; DM, diabetes mellitus; T1DM, type 1 diabetes mellitus; T2DM, type 2 diabetes mellitus; FVB, Friend virus B; CMs, cardiomyocytes; NRCMs, neonatal rat cardiomyocytes; TAX1BP1, Tax1 binding protein 1; SFA, saturated fatty acid; mM, mmol/L; HG, high-glucose; STZ, streptozocin; NMCMs, neonatal mice CMs; AFCMs, Adult feline CMs; AMCMs, Adult mice CMs; Ole, Oleate; Pal, Palmitate; NRVCs, Neonatal rat ventricular cardiomyocyte; BRD4, Bromodomain-containing protein 4; SD, Sprague Dawley; NA, not available.*

Studies of mitophagy in DCM associated with T2DM have yielded quite different results. In contrast to DCM in T1DM, cardiac lipotoxicity is a key pathologic mechanism in DCM in T2DM. Some previous studies have shown that cardiac autophagy and mitophagy is inhibited in high-fat diet (HFD)-fed mice (Sciarretta et al., 2012; Guo et al., 2013; Mu et al., 2020; Sun et al., 2020; Yu et al., 2021), but others have shown the opposite. Mellor et al. (2011) and Tang et al. (2015) found that cardiac autophagy and mitophagy are activated in the hearts of mice and rats with T2DM, respectively, which they stated would be harmful. The feeding of specific HFDs yielded the same results. For example, a diet rich in saturated fatty acids induced cardiac hypertrophy and left ventricular systolic and diastolic dysfunction in mice, but autophagy was activated (Russo et al., 2012). However, it remains controversial whether the activation of mitophagy in DCM in individuals with T2DM is beneficial or detrimental. Recently, Tong et al. (2019) found that cardiac autophagy in HFD-fed mice peaks at 6 weeks and subsequently declines, whereas mitophagy is activated for 2 months. Additionally, parkin knockout mice fed an HFD had worse cardiac function than wild-type (WT) mice (Tong et al., 2019). This implies that the activation of autophagy and mitophagy in the early stages of DCM in T2DM may have a compensatory protective effect. The discrepancies in these previous findings may be explained by differing methods of identifying mitophagy and the use of different *in vitro* and animal models of T2DM, but the underlying mechanisms require further clarification.

### Signaling Pathways Involved in Mitophagy in Diabetic Cardiomyopathy

The pathophysiologic process of mitophagy can be divided into three stages: the initiation of mitophagy, the formation of mitochondrial autophagosomes, and the degradation of the mitochondrial autophagosome. Multiple signaling pathways have been reported to be involved in these processes. Briefly, the AMPK-mTOR, insulin signaling, and Wnt/β-catenin pathways are involved in the initiation of mitophagy; the PINKl/parkin, FUNDC1, and BNIP3/NIX pathways have been reported to be associated with the formation of mitochondrial autophagosomes; and the Wnt/FZD5/calcineurin pathway has been reported to regulate the degradation of the mitochondrial autophagosome.

#### AMPK-mTOR

**FIGURE 3:**
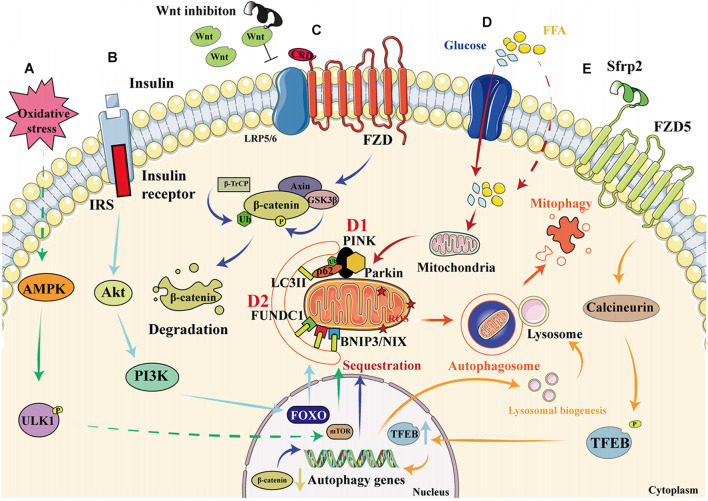
Signaling pathways that regulate mitophagy in diabetic cardiomyopathy. **(A)** When cardiomyocytes are depleted of energy, mTORC1 dissociates from the ULK1 kinase complex, ULK1 autophosphorylation increases, and this kinase phosphorylates Atg13 and FIP200. In addition, ULK1 is phosphorylated by AMPK, which induces autophagy. **(B)** When insulin binds to the insulin receptor, an IRS is recruited, which permits the docking of PI3K and the consequent activation of Akt, resulting in greater transcription of FOXO and the upregulation of autophagy. **(C)** Wnt inhibition prevents its binding to FZD, resulting in the phosphorylation of β-catenin by GSK3β, which leads to increases in the ubiquitination and degradation of β-catenin, thereby reducing its nuclear translocation and activating autophagy. **(D1)** High glucose and FFA concentrations cause mitochondrial damage and a reduction in mitochondrial membrane potential. The degradation of PINK is reduced, resulting in the recruitment of parkin to the damaged mitochondria and the ubiquitination of mitochondrial membrane proteins. This process is recognized by P62, which binds to LC3. As a result, the damaged mitochondria are anchored to the autophagic vesicle membrane, which initiates mitophagy. **(D2)** In addition to the PINK/parkin pathway, the direct binding of FUNDC1 and BNIP3/NIX proteins to LC3 on the mitochondrial membrane also initiates mitophagy. **(E)** By binding to FZD5, Sfrp2 activates calcineurin to dephosphorylate TFEB. This causes greater nuclear translocation of TFEB, resulting in the transcription of autophagy genes and an increase in lysosomal activity, which promotes the formation and degradation of the autophagosome.

AMP-activated protein kinase is a conserved cellular energy sensor that maintains energy homeostasis through the regulation of glucose and lipid metabolism (Zhao and Klionsky, 2011). This kinase couples cell growth with environmental nutrient availability, and dysregulation of the pathway underlies disease states such as cancer, cardiovascular disease, DM, and other metabolic syndromes (Herrero-Martín et al., 2009). The mechanism whereby the AMPK-mTOR pathway regulates mitophagy is presented in Figure 3A. ULK1, a mammalian homolog of the yeast protein kinase Atg1, is a conserved substrate of AMPK and is required for autophagy. In mammals, the loss of AMPK or ULK1 results in aberrant accumulation of the autophagy adaptor p62 and defective mitophagy. Under nutrient-rich conditions, mTORC1 binds to the ULK1 kinase complex, which inhibits autophagy by phosphorylating ULK1 and Atg13. When cells are starved or energy-depleted, mTORC1 dissociates from the complex, ULK1 autophosphorylation increases, and the kinase phosphorylates Atg13 and FIP200. ULK1 can then bind to and be phosphorylated by AMPK, resulting in the induction of autophagy (Egan et al., 2011).

A previous study has reported that the phosphorylation of ULK1 by AMPK is essential for exercise-induced mitophagy in mice (Laker et al., 2017). Recently, Seabright et al. (2020) found that AMPK activation promotes mitophagy by increasing mitochondrial fission and autophagosomal engulfment, without a requirement for the PINK1-parkin pathway. AMPK-mediated mitophagy in DCM has also been demonstrated in other studies. For example, helix B surface peptide (HBSP) ameliorates DCM *via* AMPK-dependent autophagy (Lin et al., 2017). Mitochondrial aldehyde dehydrogenase (ALDH2) protects against T1DM-induced myocardial dysfunction, possibly through the AMPK-dependent regulation of autophagy (Guo et al., 2015). Furthermore, mTOR has an indispensable role in the autophagy pathway. Yu et al. (2018) revealed that GLP-1 analogs reverse the high glucose-induced over-activation or phosphorylation of mTOR and ULK1 to favor the induction of autophagy. Mangiferin can increase autophagic flux to produce cardioprotective effect, which may be mediated through a decrease in mTOR phosphorylation and the consequent suppression of downstream mTORC1 signaling (Hou et al., 2018). In fact, AMPK and mTOR may co-regulate autophagy to ameliorate DCM. Metformin activates AMPK, which improves autophagy *via* inhibition of the mTOR pathway and reduces pyroptosis in DCM (Yang et al., 2019). Additionally, liraglutide ameliorates myocardial injury in DM by promoting AMPK-mTOR mediated autophagy in a rat model of DM (Zhang Y. et al., 2017).

#### Insulin Signaling

Insulin resistance is a key pathophysiologic defect in T2DM, and autophagy and mitophagy are involved in both metabolic regulation and insulin action. The relationship between autophagy, mitophagy, and insulin resistance has been previously documented (Goodpaster, 2013; Montgomery and Turner, 2015; Wanagat and Hevener, 2016). Damage to mitochondria is associated with a reduction in insulin sensitivity, and in particular, defective mitophagy leads to the accumulation of damaged mitochondria, causing the generation of large amounts of ROS, which activate the NLRP3-ASC-caspase 1 pathway, inducing insulin resistance (Yang et al., 2014). Conversely, normal mitophagy ameliorates DM by reducing the number of impaired mitochondria and restoring insulin sensitivity. Insulin signaling through the IRS-PI3K-AKT-FOXO pathway is involved in the regulation of both metabolism and autophagy (Cheng et al., 2010). Autophagy is regulated through ULK1, which is inhibited by AKT and mTORC1, and activated by AMPK (Chatterjee and Mudher, 2018; Hirata et al., 2018). Additionally, insulin mediates the normal suppression of autophagy in early life (Riehle et al., 2013). Other proteins that regulate mitophagy can also be found in the insulin signaling pathway. For example, the inhibition of Miro1 impairs mitophagy and β-cell function in T2DM, which impairs insulin signaling by inhibiting the IRS-AKT-FOXO1 pathway, leading to a reduction in glucose tolerance in diabetes (Chen et al., 2017). Furthermore, adipocyte autophagy in T2DM occurs secondary to a reduction in mTOR and insulin signaling: in patients with T2DM, a poorer response to IRS1 results in mitochondrial damage and the upregulation of autophagy, and this is associated with mTORC1 inactivation (Ost et al., 2010). The role of the insulin signaling pathway in mitophagy is described in Figure 3B.

#### Wnt/β-Catenin

The canonical Wnt/β-catenin pathway regulates stem cell pluripotency and cell fate during development (Steinhart and Angers, 2018). This involves the binding of the Wnt ligand (a secreted glycoprotein) to frizzled receptors and LRP5/6 to form a large complex at the cell surface that inhibits phosphorylation by GSK3-β, resulting in less degradation of ubiquitinated β-catenin. This results in the accumulation of β-catenin in the cytoplasm and its translocation to the nucleus, where it binds to LEF/TCF and regulates the expression of Wnt pathway target genes, such as c-myc and cyclin D1 (Janda et al., 2017). GSK-3β is involved in glucose metabolism and is dysregulated in metabolic diseases (Nusse and Clevers, 2017), and the Wnt/β-catenin pathway is activated in metabolic diseases such as diabetes (Chen et al., 2018). In DM, the accumulated intracellular ROS might divert the limited pool of β-catenin from TCF/LEF to FOXO-mediated transcription, leading to a decrease in insulin (Essers et al., 2005; Manolagas and Almeida, 2007).

Interestingly, the Wnt/β-catenin pathway might also be involved in autophagy. The activation of the Wnt pathway in various tumor cells causes β-catenin molecules to translocate to the nucleus and combine with LEF/TCF, which inhibits autophagy genes. When the Wnt pathway is inhibited, β-catenin is degraded and the expression of the autophagy genes increases, promoting autophagy, and ameliorating cellular metabolic disorders (Ziegler et al., 2018). The knockdown of Peter Pan (*PPAN*), a downstream gene in the Wnt signaling pathway that is expressed in mice and *Xenopus laevis*, promotes recruitment of the E3-ubiquitin ligase parkin to damaged mitochondria and reduces mitochondrial mass in parkin-expressing cells, which implies that Wnt signaling may regulate mitophagy (Dannheisig et al., 2019). Furthermore, vitamin D3 upregulates autophagy by inhibiting the β-catenin/TCF4/mTOR signaling pathway, thereby improving cardiac function in DCM (Wei et al., 2017). A previous study showed that activation of the nuclear β-catenin/c-Myc axis is responsible for the oxidative cardiac impairment in DCM (Liu et al., 2017). However, the results of other studies have suggested that interventions using canonical Wnt ligands do not ameliorate the cellular dysfunction caused by disorders of glucose and lipid metabolism (Zhang T. et al., 2017). Thus, suppression of the Wnt/β-catenin pathway seems to upregulate autophagy and thereby ameliorate DCM. However, it remains unclear whether it can similarly upregulate mitophagy. The proposed role of the Wnt/β-catenin pathway in mitophagy is shown in Figure 3C.

#### PINKl/Parkin

PTEN-induced kinase 1/parkin signaling is the best-characterized direct regulator of mitophagy. PINK1 is a cytoplasmic serine/threonine kinase that is degraded rapidly by mitochondrial proteases in the inner mitochondrial membrane (IMM; Greene et al., 2012). Parkin is an E3 ubiquitin ligase that ubiquitinates proteins in the outer mitochondrial membrane (OMM), thereby promoting autophagosome recruitment (Geisler et al., 2010). When mitochondria are damaged, PINK1 accumulates and recruits parkin to OMM. The IMM is depolarized and parkin ubiquitinates membrane proteins, causing their recognition by P62, a polyurethane binding protein that is degraded by autophagy. P62 binds to LC3 *via* the LC3-acting region (LIR), thereby connecting to the autophagic vesicle membrane, which induces mitophagy (Callegari et al., 2017). The effect of the PINK1/parkin pathway on mitophagy is shown in Figure 3D.

Relationships between the PINK1/parkin pathway, mitophagy, and DCM have been shown in many previous studies. In the hearts of mice with T1DM, Xu et al. (2013) found that the expression of parkin and PINK1 was lower, which implies that mitophagy is impaired in diabetes. Hydrogen sulfide promotes mitophagy in the diabetic heart by increasing the S-sulfhydration of USP8, which increases the deubiquitination of parkin (Sun et al., 2020). Sirt3 overexpression activates mitophagy to reduce myocardial apoptosis *in vitro*. However, Sirt3 silencing reduces FOXO3A deacetylation and parkin expression in diabetes. These results suggest that the Sirt3 can activate mitophagy through FOXO3A-parkin pathway to alleviate DCM (Yu et al., 2017). Mst1 also inhibits Sirt3 expression, thereby downregulating parkin and contributing to DCM (Wang et al., 2019). Furthermore, melatonin increases parkin-mediated mitophagy by suppressing Mst1 to ameliorate DCM (Wang et al., 2018). These studies provide direct evidence for the protective role of mitophagy in DCM.

#### FUNDC1

FUNDC1 is a mitophagy receptor that is located on the OMM (Morales et al., 2019) and binds to LC3 to initiate mitophagy in mammalian cells. Recently, it was demonstrated that the interaction of FUNDC1 with dynamin-related protein 1 (DRP1) and Optic Atrophy 1 (OPA1) coordinates mitochondrial fission, fusion, and mitophagy to sustain mitochondrial quality control (Chen et al., 2016). Furthermore, defective FUNDC1 contributes to the dysregulation of mitochondrial quality control in metabolic disorders (Wu H. et al., 2019). Ren et al. (2020) suggested that the loss of function of FUNDC1 inhibits mitophagy and causes deterioration in cardiac function in DCM. In diet-induced obesity, FUNDC1 was found to regulate mitochondrial quality and affect metabolism *via* MAPK signaling (Wu H. et al., 2019). However, it has also been shown that a decrease in FUNDC1 expression relieves calcium overload in mitochondria, thereby ameliorating diabetic heart disease (Wu S. et al., 2019).

In conclusion, the FUNDC1-mediated upregulation of mitophagy ameliorates DCM, but FUNDC1 may also worsen DCM through other mechanisms, such as mitochondrial calcium overload. Further studies should be conducted to determine whether DCM can be ameliorated *via* FUNDC1.

#### BNIP3 and NIX

BNIP3, a Bcl-2 family protein, was first identified in a screen for adenovirus E1B-19 K-interacting proteins and promotes cell death (Boyd et al., 1994). NIX, a homolog of BNIP3, is a transmembrane protein that is located on the OMM (Zhang and Ney, 2011). BNIP3 and NIX directly regulate mitophagy through binding to LC3II and gamma-aminobutyric acid receptor-associated protein, respectively (Schwarten et al., 2009; Hanna et al., 2012). To date, few studies have examined the effects of BNIP3 and NIX on mitophagy in DCM. However, in prediabetic male rats, the expression of BNIP3 is low, and this is associated with early changes in mitophagy and diastolic dysfunction (Koncsos et al., 2016). In contrast, another study showed that miR-133a inhibits NIX-induced mitophagy, thereby improving mitochondrial function in rodents with gestational diabetes (Mughal et al., 2015). However, whether the mitophagy induced by BNIP3 and NIX is beneficial or harmful requires further investigation. The effects of FUNDC1, BNIP3, and NIX on mitophagy are shown in Figure 3D.

#### Wnt/FZD5/Calcineurin

Calcineurin is a heterodimer that is composed of a 60-kDa catalytic subunit and a 19-kDa regulatory subunit and plays an important role in the heart (Parra and Rothermel, 2017). In recent years, regulators of calcineurin (Rcan) have become of particular interest (Serrano-Candelas et al., 2014). To date, three types of Rcans have been identified: Rcan1, Rcan2, and Rcan3. Rcan1-1L is a subtype of Rcan1 that specifically induces mitophagy and efficient mitochondrial degradation (Ermak et al., 2012). The effects of Rcan1-1L on cardiomyocytes have been shown in previous studies. Rcan1-1L overexpression induces mitophagy, which contributes to cardiomyocyte survival under hypoxic conditions (Sun et al., 2014). Moreover, Rcan1-1L reduces Ang II-induced apoptosis by activating mitophagy in human adult cardiac myocytes, which may be explained by the effect of Rcan1-1L to inhibit calcineurin/nuclear factor of activated T cells (NFAT) signaling (Duan et al., 2015). These results suggest that the calcineurin pathway might protect the heart by increasing mitophagy. However, the relationship between calcineurin and mitophagy in DM has been little studied.

The overexpression of Rcan1 increases the methylation of the Beclin-1 gene and reduces the activation of mTOR, which causes a deficiency in Miro1-mediated mitophagy in pancreatic β cells. Thus, Rcan1 may represent a therapeutic target in T2DM because it enhances the quality of β-cell mitophagy (Li et al., 2020). A novel adipokine named secreted frizzled-related protein 2 (Sfrp2) has recently been found to activate intracellular calcineurin by binding to the FZD5 receptor in vascular endothelial cells (Peterson et al., 2017). Activated calcineurin directly promotes the dephosphorylation of transcription factor EB (TFEB), a master regulator of lysosome biogenesis and autophagy, which translocates to the nucleus and promotes the expression of autophagy genes (Medina et al., 2015). Our recent studies have also shown that sfrp2 has a beneficial effect in acute myocardial infarction and heart failure (Huang and Huang, 2020; Lin et al., 2020; Wu et al., 2020; Yang et al., 2020). However, whether sfrp2 can activate the FZD5/calcineurin pathway to upregulate mitophagy in DCM has yet to be determined. The effect of the Wnt/FZD5/calcineurin pathway on mitophagy is shown in Figure 3E.

### Interaction Between Mitochondrial Dynamics and Mitophagy in Diabetic Cardiomyopathy

Mitochondrial fusion and fission (also termed mitochondrial dynamics) can be used to repair damaged mitochondria through material exchange between damaged and healthy mitochondria (Twig et al., 2008). This is mediated by mitochondrial fusion proteins, such as mitofusin 1/2 (Mfn1/2) and optic atrophy 1(OPA1); and fission proteins, including mitochondrial fission protein 1 (Fis1), Drp1, and mitochondrial fission factor (MFF). Mfn1/2 plays a pivotal role in the fusion of the mitochondrial outer membrane and OPA1 participates in intimal fusion. Fis1 and MFF participate in the recruitment of Drp1 to initiate mitochondrial fission (Rovira-Llopis et al., 2017). Mitophagy plays a key role in the coordination of mitochondrial dynamics and quality control in mitochondria. When a healthy mitochondrion fuses with a damaged mitochondrion, mitophagy is used to remove the larger, damaged mitochondrion, thereby reducing the production of ROS. Furthermore, when the damaged mitochondrion separate the damaged part by mitochondrial fission, mitophagy is used to clear the damaged part and maintain mitochondrial homeostasis (Dorn and Kitsis, 2015).

**FIGURE 4:**
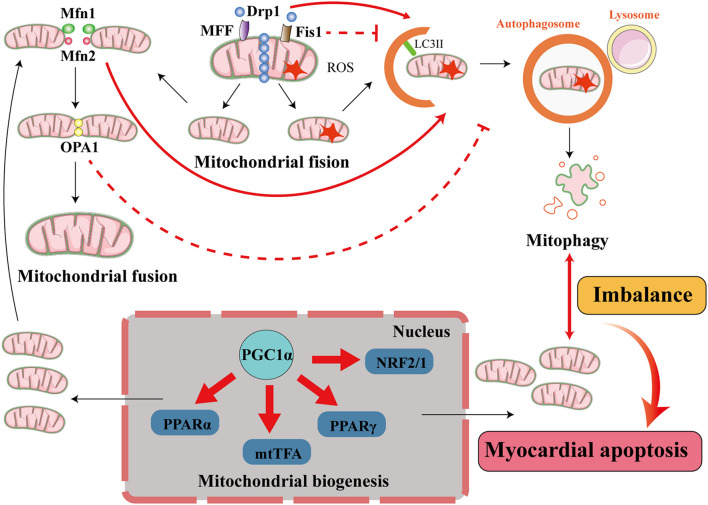
Interactions among mitophagy, mitochondrial dynamics, and mitochondrial biogenesis in diabetic cardiomyopathy. The processes of mitochondrial quality control include mitochondrial dynamics (mitochondrial fusion and mitochondrial fission), mitophagy, and mitochondrial biogenesis. In cardiomyocytes, when mitochondria are damaged, the damaged part is removed through mitochondrial fission. This process is principally mediated by fission proteins such as Drp1, MFF, and Fis1. MFF and Fis1 recruit Drp1 to promote mitochondrial fission, and Drp1 activates mitophagy but Fis1 inhibits mitophagy. The damaged mitochondrion divides into a healthy mitochondrion and a damaged mitochondrion. The former participates in other processes, such as mitochondrial fusion, which is accomplished by the fusion proteins Mfn1/2 and OPA1. Mfn2 activates mitophagy but OPA1 inhibits mitophagy, and the damaged mitochondrion is eliminated by mitophagy. When the damaged mitochondria have been cleared by mitophagy to a certain extent, the process of mitochondrial biogenesis generates new mitochondria to supplement the mitochondrial pool. This involves transcription factors such as PPARα, mtTFA, PPARγ, and NRF2/1, and cofactors such as PGC1α, and maintains the supply of energy to the myocardium. When the balance between mitophagy and mitochondrial biogenesis is lost, cardiomyocytes are damaged.

The ablation of Mfn2 in mouse cardiomyocytes prevents the depolarization-induced translocation of parkin to the mitochondria and suppresses mitophagy (Chen and Dorn, 2013), and the downregulation of Mfn2 causes an imbalance in mitochondrial dynamics, which leads to mitochondrial dysfunction and ultimately contributes to the development of DCM (Hu et al., 2019). Disruption of Drp1 induces mitochondrial elongation, inhibits mitophagy, and causes mitochondrial dysfunction, thereby promoting cardiac dysfunction (Ikeda et al., 2015). These findings imply that mitochondrial dynamics and mitophagy ameliorate DCM, but others have made findings that were not consistent. For example, SIRT4 interacts with OPA1 to promote mitochondrial fusion and inhibit mitophagy (Lang et al., 2017). Xian et al. (2019) demonstrated that the SNARE protein syntaxin 17 (STX17) initiates mitophagy upon the depletion of Fis1, indicating that proteins involved in mitochondrial dynamics may inhibit mitophagy and cause deterioration in DCM. Therefore, complex interactions may be involved in mitochondrial dynamics and mitophagy in DCM, depending on the exact pathophysiology and environment present (Figure 4).

### Balance Between Mitophagy and Mitochondrial Biogenesis in Diabetic Cardiomyopathy

Mitophagy is involved in mitochondrial homeostasis in cardiomyocytes and has a protective effect in DCM. However, in some conditions, the activation of autophagy does not improve cardiac function and may facilitate cardiomyocyte death (Mizushima and Komatsu, 2011). The unique mechanism of autophagy-induced cell death is known as autosis (Liu et al., 2013). In mice with T1DM, the upregulation of autophagy aggravates cardiac dysfunction (Xu et al., 2013). AKT2 deficiency activates mitophagy and causes cell death because of the excessive removal of mitochondria (Santi and Lee, 2011). These results suggest that excessive upregulation of mitophagy may not ameliorate DCM, but rather worsen heart failure. Mitochondrial biogenesis is a process that generates new mitochondria to replenish the mitochondrial pool and occurs alongside mitophagy. Excessive mitophagy destroys mitochondria in myocardial cells, resulting in insufficient myocardial energy supply, while mitochondrial biogenesis supplements the healthy mitochondria and improves cardiac function. Therefore, a balance between mitochondrial biogenesis and mitophagy is essential for cellular metabolic homeostasis (Potes et al., 2019).

Mitochondrial proteins are encoded by both the nuclear and mitochondrial genomes, which are synchronized by peroxisome proliferator-activated receptor gamma coactivator 1-alpha (PGC-1α). PGC-1α is a master regulator that activates and coordinates mitochondrial biogenesis through its effects on multiple transcription factors, including PPARγ and PPARα, estrogen receptor-related α (ERRα), nuclear respiratory factors 1 and 2 (NRF1/2), and mitochondrial transcription factor A (mtTFA; Fernandez-Marcos and Auwerx, 2011). Mitochondrial biogenesis is upregulated in the diabetic heart (Shen et al., 2004; Duncan et al., 2007) and this may compensate for excessive mitophagy. An imbalance between mitophagy and mitochondrial biogenesis may lead to insufficient energy supply to the myocardium in DCM. Indeed, it has been suggested that mitophagy is balanced by mitochondrial biogenesis to mitigate mtDNA damage and improve the outcomes of ischemia/reperfusion stress (Andres et al., 2017). However, the mechanism whereby mitophagy and mitochondrial biogenesis can be balanced during DCM remains to be determined. The relationship between mitophagy and mitochondrial biogenesis is illustrated in Figure 4.

### Mitophagy as a Therapeutic Target in Diabetic Cardiomyopathy

Improvement in mitochondrial function is a therapeutic goal in DCM. Mitochondrial function can be restored using various methods, such as inducing uncoupling (Cadenas, 2018), administering antioxidants (Ni et al., 2016), and administering biofuels (Gollihue and Rabchevsky, 2017). However, clinical trials have shown no effects of antioxidant therapies on heart failure in patients with DM, which suggests that the use of antioxidants to antagonize existing ROS is insufficient to reduce diabetic cardiac injury (Lonn et al., 2002; Johansen et al., 2005). The stimulation of mitochondrial uncoupling may reduce mitochondrial superoxide production but would also reduce mitochondrial oxidative efficiency in the diabetic heart (Boudina and Abel, 2006). Mitochondrial transplantation, in which dysfunctional mitochondria are replaced by healthy mitochondria, is a novel therapeutic approach for ischemia/reperfusion related disorders (Emani et al., 2017; McCully et al., 2017), and has been shown to improve the post-ischemic recovery of myocardial function and reduce myocellular injury in the diabetic heart (Doulamis et al., 2020). It has also been suggested that mesenchymal stem cells have regenerative abilities in organs including the heart that are based upon mitochondrial transfer. However, it has not been determined whether this approach would also be effective in DCM (Paliwal et al., 2018). Finally, although mitophagy represents a potential therapeutic target and has been tested in preclinical studies, no data have been collected in humans.

Some existing drugs, including metformin, pioglitazone, exendin-4 (a GLP-1 receptor agonist), angiotensin receptor blockers, and resveratrol, may affect mitophagy (Kubli and Gustafsson, 2015; Wassef et al., 2018). Of these, the effect of metformin, a first-line antidiabetic medicine, to improve mitophagy in DCM has attracted great interest. Metformin activates AMPK, and can therefore stimulate autophagy and protect the heart against DCM (Sasaki et al., 2009). Furthermore, long-term AMPK activation by metformin has been shown to prevent cardiomyopathy by upregulating autophagy in diabetic OVE26 mice (Xie et al., 2011). However, the effect of metformin on cardiovascular mortality of T2DM patients remains controversial. A recent meta-analysis suggests that compared with untreated T2DM patients, metformin-treated patients was not associated with lower risk of all-cause mortality, cardiovascular mortality, and heart failure (Li et al., 2021). Therefore, whether the mechanism of metformin improving myocardial damage through mitophagy in diabetic animal models is also applicable to T2DM patients still needs to be supported by more clinical studies.

## Summary and Future Perspectives

It is now clear that mitophagy plays a central role in the regulation of mitochondrial quality control in DCM and that the regulation of mitophagy represents a promising potential therapeutic strategy for this complication. However, several outstanding issues remain in this field of research. First, the regulation of mitophagy in DCM is complex and it is unclear how it is related to the process of mitochondrial biogenesis. For example, deleterious effects of mitophagy and autophagy in DCM have been reported (Xu et al., 2013). The deterioration in cardiac function that is caused by mitophagy might be the result of insufficient energy supply to cardiomyocytes. Therefore, the re-establishment of the balance between mitophagy and mitochondrial biogenesis might be the most appropriate aim in DCM. It has been shown that both mitophagy and mitochondrial biogenesis occur during cardiac surgery involving cardiopulmonary bypass, and it has been suggested that the mitophagy is balanced by mitochondrial biogenesis during the ischemia/reperfusion stress that is experienced during surgery (Andres et al., 2017). However, it is unclear whether restoration of the balance between mitophagy and mitochondrial biogenesis would improve the prognosis in DCM. Second, a number of signaling pathways have been identified that regulate mitophagy, including PINK1/parkin, AMPK-mTOR, and the Wnt pathway. However, their interactions in the regulation of mitophagy are not yet fully understood. Third, the roles of mitophagy in T1DM and T2DM remain controversial, because inconsistent findings have been made, which may relate to differences in pathophysiological mechanisms, and/or the animal models and means of evaluating autophagy that were used. Future studies should explore whether mitophagy plays differing roles in T1DM and T2DM and the mechanisms involved.

## Author Contributions

HZ wrote the first draft of the manuscript. All authors provided editing assistance, and read and approved the final manuscript.

## Conflict of Interest

The authors declare that the research was conducted in the absence of any commercial or financial relationships that could be construed as a potential conflict of interest.

## Publisher’s Note

All claims expressed in this article are solely those of the authors and do not necessarily represent those of their affiliated organizations, or those of the publisher, the editors and the reviewers. Any product that may be evaluated in this article, or claim that may be made by its manufacturer, is not guaranteed or endorsed by the publisher.

## Abbreviations

DM: diabetes mellitus
DCM: diabetic cardiomyopathy
ATP: adenosine triphosphate
ROS: reactive oxygen species
IRS1/2: insulin substrate receptor 1/2
PINK1: PTEN-induced kinase 1
Rab9: Ras-like proteins in brain 9
HFD: high-fat diet
AMPK: AMP-activated protein kinase
mTOR: mechanistic target of rapamycin kinase
ULK1: unc-51 like autophagy activating kinase 1
FIP200: focal adhesion kinase family interacting protein of 200 kD
LRP5/6: low-density lipoprotein receptor-related protein 5/6
GSK3-β: Glycogen synthase kinase-3
LEF/TCF: Lymphoid Enhancer Factor 1/T Cell Factor 1
IMM: inner mitochondrial membrane
OMM: outer mitochondrial membrane
USP8: The deubiquitinating enzyme ubiquitin-specific protease 8
FZD5: frizzled-5
Rcan: calcineurin
NFAT: nuclear factor of activated T
Sfrp2: secreted frizzled-related protein 2
TFEB: transcription factor EB
Mfn1/2: mitofusin 1/2
OPA1: optic atrophy
Fis1: fission protein 1
MFF: mitochondrial fission factor
PI3K: PI3 kinase
PGC-1 α: peroxisome proliferator-activated receptor gamma coactivator 1-alpha
PPAR γ: peroxisome proliferator-activated receptor gamma
PPAR α: peroxisome proliferator-activated receptor αα
ERR α: estrogen receptor-related αα
NRF1/2: nuclear respiratory factors 1 and 2
mtTFA: mitochondrial transcription factor A.
